# Alpine skiing injuries in Finland – a two-year retrospective study based on a questionnaire among Ski racers

**DOI:** 10.1186/2052-1847-6-9

**Published:** 2014-02-24

**Authors:** Antti J Stenroos, Lauri E Handolin

**Affiliations:** 1 Department of Orthopedics and Traumatology, Helsinki University Hospital, PO Box 266, Helsinki, HUS FI-00026, Finland

## Abstract

**Background:**

Alpine skiing is one of the most popular winter sports in the world. Nevertheless, it has always been associated with a high risk of injury. There are however, only a few studies that have examined the risk of injury of competitive skiers, especially of the junior ski racers.

**Methods:**

The inclusion criterion was an injury in alpine skiing resulting in a pause in training longer than one week. Athletes of all ages were included. The study period was from the start of the season of 2008–2009 to end of the season of 2009–2010 (two years).

**Results:**

The average annual number of ski racers in Finland was 661. There were 61 injuries (36 males with a median age of 14 years, 25 females with a median age of 14) fulfilling the inclusion criteria. Ligamentous knee injury was the most frequent (17) and lower leg fracture the second common (16) injury, respectively. There was a female dominance in the ACL injuries. Only one major abdominal injury and no major head injuries were observed. The overall training pause was 26 weeks after the ACL injury and 17 weeks after the lower leg fracture, respectively.

**Conclusion:**

The most common and most disabling injuries affect the knee and the lower leg. The high number of lower leg and ACL injuries is alarming. A continuous and careful monitoring of injuries needs to be established to assess this trend. A systematic review of injuries is the appropriate way to monitor the effects of changes made in terms of safety. The present retrospective two-year pilot study forms a base for a continuous alpine ski injury survey in Finland.

## Background

Alpine skiing is one of the most popular winter sports in the world even though it has always been associated with a high risk of injury. Previous studies have shown that the overall injury rate among recreational skiers is approximately three injuries per 1,000 skier days [1-3]. However, there are only a few studies that have examined the risk of injury of competitive skiers, especially of the junior ski racers. There are some differences in the overall injury patterns and incidents between ski racers and recreational skiers. In studies of single events the incidence was estimated to be 1.9-4 per 1000 runs at the competitive level [4,5]. Older studies have shown that 72–83% of ski racers have had at least one serious injury during their career [6,7]. Flørenes et al. found the injury rate in the FIS World Cup 2006–2008 to be as high as 36.7 per 100 athletes [8].

The types of injuries related to alpine skiing have changed over time, as the equipment and slope-grooming technologies have evolved. It can be assumed that changes in ski equipment and modern slope-grooming techniques have led to reductions in the overall injury rate, especially for ankle fractures and tibia fractures [1,9-12]. The knee has been reported as the most common site of injury, accounting for 20-27% of all skiing injuries [1-3,9-12].

Even though the ski equipment has undergone great changes during the last ten years, ACL injury rates among competitive alpine skiers are high and have not declined in the last years [13]. With the new type of skis, the "Slip and catch" mechanism (results in flexion and internal rotation) of the ACL injury has become the most common ACL injury mechanism among competitive skiers [14]. The general consensus is that the flexion-internal rotation injury mechanism is especially related to the skiing equipment [15]. The International Skiing Federation (FIS) published new ski regulations before the start of season 2012–2013. In the regulations the turning radius and the aggressiveness of the skies are to be decreased, both of which are risk factors in ACL injuries [16].

There is a need for a national injury surveillance system in terms of continuous assessment in injury incidence and prevention. However, alpine skiing injuries as well as injury rates and patterns have not yet been extensively studied in Finland. The aim of the present study was to retrospectively investigate the injury patterns and rates in Finland at the competition level of alpine skiing taking place both in races and in trainings, and to pilot a continuous competition alpine skiing injury survey in Finland.

## Methods

Injuries among ski racers of all ages, from children to adults, taking place in alpine skiing competitions or snow training sessions in slopes in Finland were retrospectively studied. The study period was from the start of the season of 2008–2009 to the end of the season of 2009–2010. The inclusion criterion was an acute injury resulting in a training pause longer than one week in an athlete having a competitor’s license registered under the national alpine ski federation (Ski Sport Finland). Injuries in recreational skiers were excluded resulting in a clear focus on ski racing and training. Also repetitive strain injuries were excluded from the present study.

The identification of the injured athletes fulfilling the inclusion criteria was conducted by repeated e-mail enquiries and personal contacts with all ski clubs in Finland several times during the study period. The purpose and procedures of the study were explained at the team captain’s meetings in the beginning of the season 2010–2011, where representatives from all ski clubs were present. At these meetings, the coaches were asked to inform their athletes and the guardians of the study. The authors have close contacts with all ski clubs in Finland and the head coaches were contacted regularly in order not to miss any possible injuries fulfilling the inclusion criteria.

After the identification of an athlete fulfilling the inclusion criteria, the data collection was conducted directly from the athletes (or from their guardians in case of younger persons) by personal communication and by a standardized written questionnaire. Most of the athletes were interviewed, and the questionnaire filled, at the skiing events in person. If the athlete was not met, the written questionnaire was sent by mail.

Collected data consisted of patient characteristics, mechanism of the skiing accident, use of protective gear, nature of injuries, as well as the specific injury diagnosis, given care, the length of recovery off-ski period, and a subjective outcome at six months post injury. The care given and the specific ICD-10 diagnosis was verified by review of medical records in almost all of the cases. The subjective outcome was assessed by a five-step scale; no, mild, moderate, major or severe (not able to ski) discomfort in skiing at six months post injury. Descriptive statistics is presented as mean and standard deviations. Categorical data is presented by frequency counts, proportions and percentages. To compare gender and age, the chi-square test was used for categorical variables. P values for the comparisons are presented.

The study protocol was approved by the review board of Helsinki University Central Hospital.

## Results

During the study period of two years, on average 661 athletes of all ages took part in alpine ski races annually in Finland. There were a male dominance (65%) compared to females (35%). Most of the active alpine ski competitors are adolescents in Finland; 82% were aged between nine and 15 years (adults 18%).

There were 61 injuries (36 male and 25 female) fulfilling the inclusion criteria during the study period (26 injuries in the season of 2008–2009 and 35 in the season of 2009–2010). The mean age of the injured females was 14 years (range 10–36 years, SD 3,1) and of the males 14 years (range 9–32 years, SD 4,9), respectively. There were no difference in injury incidense between males and females.

The majority of accidents occurred during the winter-snow months from November to May. Only a few injuries took place in summer glacier training camps.

The most common mechanisms of injury were falling down on the same level, which includes collision with the icy surface (49%), collision on an on-piste area (30%), and collision on an off-piste area, collisions on-piste and off-piste areas includes collisions with an immovable object or a another person (8%). Most of the injuries took place in giant slalom (GS) (56%). Nineteen (31%) injuries occurred in slalom (SL) and eight (13%) in super giant slalom (SG) (Table 1).

**Table 1:** Occured injuries by discipline and the mechanism of injury

**Mechanism of injury**	**Slalom (SL)**	**Giant slalom (GS)**	**Super giant slalom (SG)**
	***Males (mean age)/females (mean age)***	***Males (mean age)/females (mean age)***	***Males (mean age)/females (mean age)***
Falling on the same level	6 (13)/3 (14)	9 (14)/9 (14)	1 (12)/2 (13)
Collision; on-piste area	3 (12)/5 (16)	6 (15)/2 (14)	2 (15.5)/0
Collision; off-piste area	0/0	2 (18)/3 (11)	0/0
Loss of control in jumps	0/0	2 (13.5)/0	2 (13)/1 (21)
Distorsion without falling	1 (14)/1 (16)	1 (14)/0	0/0
TOTAL	10 (13)/9 (15)	20 (14)/14 (14)	5 (13)/3 (16)

There were no lethal injuries during the study period. Forty-five (74%) of the injuries prompted hospital admission with the median length of hospital stay of one day (range 1–21 days). The most common reason for admission was surgical operation (26 cases), non-operative management of the injury (16 cases), and post-commotion survey after a head trauma (4 cases). Only one operation was carried out due to abdominal parenchymal injury. All other operations were due to extremity injuries. Three of the operations were performed on the upper and 23 on the lower extremity injuries, respectively. Two athletes gave up their career after sustaining the injury, but only one retirement was purely due to the injury (lower leg injury) preventing alpine skiing at a competition level.

The lower extremity was the most commonly injured body area (64%). In upper extremity there were 17 injuries and in the head four injuries. There was also one abdominal injury, but no thoracic or spinal trauma was observed. More detailed information of the mechanism of injury and the injured body area is presented in Table 2.

**Table 2:** The mechanisms of injury by injured body area

**Mechanism of injury**	**Head**	**Abdomen**	**Spine**	**Upper extremity**	**Lower extremity**
	***Males (mean age)/females (mean age)***	***Males (mean age)/females (mean age)***	***Males (mean age)/females (mean age)***	***Males (mean age)/females (mean age)***	***Males (mean age)/females (mean age)***
Collision; on the piste area	1 (12)/0	0/0	0/0	4 (13.5)/3 (13)	6 (14)/4 (15)
Collision; off piste area	0/0	1 (22)/0	0/0	1 (14)/2(14.5)	0/1 (14)
Loss of control in jump	2 (12)/-	0/0	0/0	2 (13.5)/0	0/1 (21)
Falling on the same level	1 (12)/-	0/0	0/0	3 (14)/2 (14)	12 (13)/12 (15)
Distorsion without falling	0/0	0/0	0/0	0/0	2 (14)/1 (16)
TOTAL	4 (12)/0	1 (22)/0	0/0	10 (14)/7 (14)	20 (14)/19 (15)

Seventeen (28%) of all the injuries were in the upper extremity. Nearly all of them were fractures, only one being a glenohumeral dislocation. The most common injury was a fracture in the hand (including fingers). No distal radial fractures were seen. All, but one thumb fracture, were treated non-operatively. The incidence of upper extremity and hand injuries was similar between males and females. None of the skiers with an upper extremity injury were sustaining greater than mild discomfort in skiing six months after the injury. The average length of recovery off-ski period was 51 days (range 7–120 days). More detailed information of the upper extremity injuries is presented in Table 3.

**Table 3:** Detailed information of the upper extremity injuries in males and females

**Injury location**	**Number of injuries**	**Fracture**	**Dislocation**	**Length of recovery off-ski period in days**
	***Males (mean age)/females (mean age)***	***Males (mean age)/females (mean age)***	***Males (mean age)/females (mean age)***	***Males/females***
Collar bone	1 (12)/1 (12)	1 (12)/1 (12)	0/0	40/59
Glenohumeral joint	1 (14)/0	0/0	1 (14)/0	40/0
Humeral bone	2 (13.5)/0	2 (13.5)/0	0/0	45 (13.5)/0
Radial/ulnar bone	0/3 (14.5)	0/2 (14.5)	0/0	0/3(60)
Hand (incl. fingers)	6 (14.5)/3 (12)	6 (14.5)/3 (12)	0/0	14 (14.5)/30 (12)

More than half of all the reported injuries located in lower extremities, 21 of them being knee injuries and 16 lower leg (tibia and fibula) fractures. The incidence of lower leg injury was significantly higher in females compared to males (P = 0.02)

Eight of the knee injuries were anterior cruciate ligament (ACL) injuries. The number of distal lower leg injuries (injuries inside the lower part of the ski boot) was low. All ACL injuries and six of lower leg fractures were treated surgically. Ligamentous knee injuries were more common in females (p = 0,02). Almost all (88%) skiers with a lower leg fracture and all skiers with ACL injure sustained greater than mild discomfort in skiing six months after the injury. The average length of the recovery off-ski period was 175 days (range 150–180 days) after the ACL injury and 115 days (range 45–180 days) after the lower leg fracture. More detailed information of the lower extremity injuries is presented in Table 4.

**Table 4:** Detailed information of the lower extremity injuries in males and females

**Injury location**	**Male/Female**	**Fracture**	**Ligament injury**	**Contusion**	**Length of recovery off-ski period in days**
	***Males (mean age)/females (mean age)***	***Males (mean age)/females (mean age)***	***Males (mean age)/females (mean age)***	***Males (mean age)/females (mean age)***	***Males/females***
Knee	11 (18)/10 (16)	0/0	8* (18)/9** (15.5)	3 (12)/1 (16)	45/180
Lower leg (tibia and fibula)	8 (14)/8 (14)	8 (14)/8 (14)	0/0	0/0	120/115
Ankle	0/2 (14)	0/1 (12)	0/1 (16)	0/0	0/45

*Two ACL injuries in males.

**Six ACL injuries in females.

## Discussion

There are some differences in the overall injury patterns between the present study conducted among ski racers and in those reported among recreational skiers; we observed fewer hand and head injuries, but the number of the lower leg (tibia and fibula) fractures was higher [1-3,17]. The low number of head injuries compared to recreational skiers can be explained both by the mandatory use of protective helmets in alpine skiing as well as by the decreased risk of colliding with another skier as there is only one skier on the course at the time versus crowded slopes in recreational skiing [18-21].

In the present study, most of the ski racers were adolescents and not competing in maximum speed discipline (downhill), which is clearly reflected in the present results the majority of the injuries being extremity injuries. In general, high-speed training and racing are done to a much lesser extent than relatively lower speed disciplines such as slalom and giant slalom. The higher the speed is and the jumps are, the higher goes the risk of major head, thoracic, abdominal or spinal injuries. Also, one has to keep in mind that we included all the injuries taking place when an athlete was on skies, not only the ones taking place in races, but also the ones taking place in trainings.

In the present study there was only one abdominal injury in giant slalom due to collision which took place outside the groomed slope area (loss of control on-piste followed by unintended deviation to off-piste). However, this injury prompted surgical intervention and, thus, indicates careful decision making in placing and putting up the safety nets. Prevention of collisions between alpine skiers and recreational skiers also needs to be stressed; training and racing areas must always be securely marked and separated.

In the present study the lower leg (tibia and fibula) fractures comprised one quarter of all the injuries, thus being more common than would have been expected based on previous reports: 5% of all injuries in recreational skiers [1-3,9-12], and 9% in adult ski racers according to the FIS injury surveillance report [22]. Florenes et al. found that lower leg injuries were the second most frequently injured body part among world cup skiers with 11,5% of all injuries [8]. However, there is one major difference in study populations between above referred studies and the present study; the median age of the patients sustaining a tibial fracture in our study was only 14 years, and 82% of all patients were under 16 years of age. The risk of a tibial fracture in skiing is reported to be four times higher for a child than for an adult [23]. In addition to that, there also is a possibility of inadequately functioning ski-binding-boot (SSB) system resulting from a inadequate high binding release values. The release of a binding is important in preventing injuries to the lower extremity [24], and this needs to be acknowledged by all the coaches. The study period covered only two seasons, which also may have resulted in statistical bias as being relative short. Since we included the injuries taking place also in trainings, not only in races, there is a possibility that slope conditions in trainings might sometimes be suboptimal due to lower number of course maintenance side-slippers compared to race conditions. This all indicates, that a continuous surveillance is mandatory to find out the trend of tibial fracture incidence in the future.

The most common problem among professional skiers is the knee injury, and the most frequent specific diagnosis is a complete rupture of the anterior cruciate ligament [8]. It has been reported that females have a higher risk of ACL injury compared to males [25-27]. The definite reasons for this are still unknown, but it has been stated that the likelihood of an ACL injury is affected by the menstrual cycle in females [27], and that the muscle strength and balance both in lower torso and extremities have a major impact on the ALC injury risk. In the present study, females accounted for one third of the study population and had three quarters of all observed ACL injuries. However, due to the low number of patients, any definite statements cannot be made concerning the ACL injury incidence between males and females, but the tendency towards females having more ACL injuries was apparent. When comparing the absolute number of injuries the overall injury risk was similar for males and females. There are some differences between males and females regarding injury location.

The retrospective nature and relatively small number of patients are the major limitations of the study. In the present retrospective survey, it cannot be ruled out that some minor injuries fulfilling the inclusion criteria might have gone unreported due to their relatively benign nature; the athletes may have either forgotten some of the injuries they have suffered during the previous seasons and/or were not willing to report on them. In major injuries, such as lower leg fractures or ACL injuries, the exposure is relatively reliable. The low number of patients may also result in bias; thus it is more sensible to draw conclusions of the tendencies than to base them on absolute numbers. The relative low number of certain injuries makes the statistical comparisons prone to bias and even impossible in some cases. Type B error has to keep on mind when interpreting the results. Consequently, the present data does not represent the solid national epidemiology as such, but it gives a fair overall picture of the presentation of the injuries among alpine ski racers, mainly juniors, in Finland.

## Conclusions

The high prevalence of lower leg injuries compared to earlier studies is alarming. The increasing number of lower leg and ACL injuries is worrying and actions should be taken to fight this trend. The ski clubs and especially trainers should be active in making sure that skiers have adequate equipment and right adjustments in the ski-binding-boot (SSB) system.

The only way to monitor the effects of changes made in terms of safety is continuous and systematic reviewing of injuries. It is necessary that is done by the official national alpine ski federation. The present retrospective two-year study will form the base for a continuous alpine ski injury survey in Finland.

## Competing interests

All authors read and approved the final manuscript.

## Authors’ contributions

AS took part in planning of the study, collected the data, and did the writing. LH was in charge of planning of the study, took part in analysis, and took part and reviewed the writing. Both authors read and approved the final manuscript.

## Pre-publication history

The pre-publication history for this paper can be accessed here:

http://www.biomedcentral.com/2052-1847/6/9/prepub
